# Surface Hardness of Resin Cement Polymerized under Different Ceramic Materials

**DOI:** 10.1155/2012/317509

**Published:** 2012-04-02

**Authors:** Pimmada Kesrak, Chalermpol Leevailoj

**Affiliations:** ^1^Department of Conservative Dentistry, Faculty of Dentistry, Prince of Songkla University, Hatyai, Songkhla 90112, Thailand; ^2^Esthetic Restorative and Implant Dentistry Program, Faculty of Dentistry, Chulalongkorn University, Henri-Dunant Road, Patumwan, Bangkok 10330, Thailand

## Abstract

*Objectives*. To evaluate the surface hardness of two light-cured resin cements polymerized under different ceramic discs. *Methods*. 40 experimental groups of 2 light-cured resin cement specimens (Variolink Veneer and NX3) were prepared and polymerized under 5 different ceramic discs (IPS e.max Press HT, LT, MO, HO, and Cercon) of 4 thicknesses (0.5, 1.0, 1.5, and 2.0 mm), Those directly activated of both resin cements were used as control. After light activation and 37°C storage in an incubator, Knoop hardness measurements were obtained at the bottom. The data were analyzed with three-way ANOVA, *t*-test, and one-way ANOVA. *Results*. The KHN of NX3 was of significantly higher than that of Variolink Veneer (*P* < 0.05). The KHN of resin cement polymerized under different ceramic types and thicknesses was significant difference (*P* < 0.05). *Conclusion*. Resin cements polymerized under different ceramic materials and thicknesses showed statistically significant differences in KHN.

## 1. Introduction

Currently, there is an increased demand for esthetic restorations, especially all-ceramic restorations, including all-ceramic crowns, inlays, onlays, and veneers. Therefore, many new ceramic systems have been developed. Current ceramic materials have numerous superior properties, such as their esthetic lifelike appearance, biocompatibility, chemical stability, and high compressive strength. Continuing development in ceramic core materials has made all-ceramic restorations a more valuable clinical option. Ceramic core materials are categorized into 3 different types: glass, alumina based, and zirconia [1].

 In addition to the physical properties of ceramics, luting materials are important for the longevity of ceramic restorations. The use of resin cement in combination with a dental adhesive will strengthen all-ceramic restorations and influence the longevity of the restoration [2]. Furthermore, resin cement has an effect on the esthetics of restorations due to the color of the cement. Because ceramic is a translucent material, using tooth-colored resin cement under ceramic restorations allows the observation of the color of the cement and improves the esthetics of the restoration. Alternatively, some resin cements can obscure dark-colored teeth and that may affect the color of restoration.

 Resin cements are composed of methacrylate or Bis-GMA, similar to resin composite. According to the activation mode, resin cements are usually divided into three groups: chemically activated (self-cured), photoactivated (light-cured), and dual-cured, in which the polymerization was affected by both chemical and light activation [3]. Light-cured resin cements use a photoinitiator, primarily camphorquinone; the polymerization process begins when activated by light from the light-curing unit. Light-cured resin cements are often preferred to chemical-cured and dual-cured resin cements because of their on-demand polymerization characteristic. Moreover, this type of resin cement allows for easier manipulation due to the lack of preparation process, such as mixing before use, which results in decreased air incorporation into the cement and also decreased color instability. However, light-cured resin cements also have a limitation associated with the polymerization process as they require sufficient light to initiate and maintain polymerization, especially in deep cavities or thick restorations that may attenuate the light from the light-curing unit. Incomplete polymerization of materials will affect both physical and biological properties, such as surface hardness, color instability, toxicity from residual monomer [4–7], and decreased bond strength between tooth and restoration (which can also decrease the longevity of the restoration).

 The effectiveness of light to initiate polymerization of resin-based materials requires the appropriate wavelength determined by the type of photoinitiator incorporated in the resin-based material [8]. Camphorquinone is effectively activated by light at a wavelength range of 375–500 nm, with a peak maximum absorption at 468–470 nm [9, 10] and also with a light intensity high enough to activate polymerization. Factors affecting light efficiency include the light-curing unit, exposure time, and any object between the light tip and the resin cement [11–13]. Recently, the light emitting diode (LED) light-curing units have become very popular among dental practitioners because of advantages over existing light-curing units. The LED produces a narrow spectrum of light that falls within the absorption spectrum of the camphorquinone [14]. Furthermore, an LED has a lower power requirement and is powered by a rechargeable battery which makes the device a cordless, portable, and lightweight unit with a longer lifespan.

 Surface hardness testing is one aspect of the polymerization measurement method [15–17]. It provides a strong correlation to the light intensity used in polymerization activation [18–20]. Hence, this study aimed to evaluate the surface hardness of two light-cured resin cements polymerized under different types and thicknesses of ceramic discs.

## 2. Materials and Methods

For IPS e.max Press (Ivoclar Vivadent, Liechtenstein) ceramic groups, the ceramic discs were fabricated from IPS e.max Press high-translucency ingot (HT-A1), low-translucency ingot (LT-A1), medium-opacity ingot (MO-0), and high-opacity ingot (HO-0), with a 10 mm diameter and thicknesses of 0.5, 1.0, 1.5, and 2.0 mm. The wax patterns, 10 mm in diameter with a level of thickness exceeding 0.2 mm each, were fabricated to the ceramic discs by the lost wax, heat press process. The ceramic discs were polished with an automatic polishing machine (DPS 3200, IMPTECH, South Africa) and silicon carbide paper nos. 400, 600, 800, and 1200, respectively. The final thickness was measured by a digital micrometer (Mitutoyo, Japan).

 For Cercon (DeguDent, Germany) ceramic groups, the ceramic discs were fabricated from a Cercon base (white) to a 10 mm diameter and thicknesses of 0.5, 1.0, 1.5, and 2.0 mm. The Cercon base was cut into the framework in a circular shape and trimmed by the silicon carbide paper. The framework required a 30% greater diameter and thickness to compensate for shrinkage during the sintering process. After sintering, the ceramic discs were polished and measured, as previously described.

 The resin cements used in this study were Variolink Veneer (Ivoclar Vivadent, Liechtenstein) shade high value +3 and NX3 Nexus Third Generation (Kerr Corporation, USA) shade white opaque. The resin cement was inserted into a black PVC mold with a centered hole 6.0 mm in diameter and 0.5 mm deep. A glass slab (0.04 mm thick) was placed above the mold and resin cement. The resin cement underwent light activation in two modes: direct light activation (control) or light activation through ceramic discs (experimental), with 5 ceramic groups and 4 thicknesses each. In the experimental groups, the ceramic discs were placed between the tip of the light guide of the light-curing unit and the glass slab covering the resin cement before activation. The resin cement was light activated by an LED light-curing unit (Demi, Kerr Corporation, USA) with an irradiance of 1,450 mW/cm^2^ for 40 seconds, in contact with the ceramic material or glass slab. The intensity of the light-curing units was measured with a hand-held radiometer (L.E.D. radiometer by Demitron, Kerr Corporation, USA) that was recalibrated after 10 times of usage. The specimens were stored in an incubator (CONTHERM 160 M, CONTHERM Scientific Ltd., New Zealand) at 37°C for approximately 24 hours. Forty two groups of 12 resin cement specimens each were tested.

The micro-hardness tester (FM-700e TYPE D, FUTURE-TECH, Japan) with 50-gram force for 15 seconds was used for Knoop hardness testing. Three indentations were made on the bottom surface of each specimen, with a 1 mm distance between indentations, and the means were then calculated. Measurements were made under 40x magnification.

**Statistical Analysis**

SPSS software version 17 was used to analyze the results at a 0.05 significance level (*P* < 0.05). The effects of resin cement types, ceramic types, and thicknesses of ceramic on resin cement hardness were analyzed using three-way ANOVA. The independent *t*-test was used to compare the 2 different types of resin cement. To compare the surface hardness of light-curing resin cement cured through different thicknesses of ceramics, we used one-way ANOVA and Tukey's multiple comparison test. Finally, the one-way ANOVA and Tukey's multiple comparison test were used to compare the differences of the resin cement surface hardness cured through different types of ceramic having the same thickness.

## 3. Results

Table 1 shows the surface hardness value of two resin cements when polymerized under different types and thicknesses of ceramic discs. The surface hardness of Variolink Veneer was statistically lower than that of NX3 in all experimental groups. The surface hardness of both resin cements (Variolink Veneer and NX3) when polymerized under ceramic discs presented statistically significant differences from the control. For Variolink Veneer, the surface hardness of resin cement polymerized under IPS e.max Press HT 2.0 mm, LT 2.0 mm, MO 1.5, 2.0 mm, HO 1.0, 1.5, and, 2.0 mm and Cercon 1.0, 1.5, and 2.0 mm was significantly lower than that of the control group. The surface hardness of NX3, polymerized under IPS e.max Press HT 2.0 mm, LT and MO at a thickness level of 1.5, 2.0 mm, and HO and Cercon at a thickness level of 1.0, 1.5, and 2.0 mm was significantly lower than that of control. Furthermore, the surface hardness of both resin cements when polymerized under each ceramic type showed statistically significant differences between thicknesses. Resin cement polymerized under 2.0 mm ceramic discs tended to have the lowest surface hardness value in each ceramic.

When comparing the surface hardness value of two resin cements polymerized under different ceramic types with the same thickness, the surface hardness of Variolink Veneer in all thicknesses of ceramic showed no statistically significant differences among all ceramic types. The resin cement polymerized under IPS e.max Press HT and Cercon tended to have the highest and lowest surface hardness, respectively, through each thickness range. For NX3, the surface hardness of cement polymerized under 0.5 mm ceramic discs showed no statistically significant differences among all ceramic types. When NX3 was polymerized under 1.0, 1.5, and 2.0 mm ceramic discs, the surface hardness of resin cement under IPS e.max Press HT was significantly higher than Cercon. However, when NX3 polymerized under 2.0 mm ceramic discs, the surface hardness of resin cement under both IPS e.max Press HT and MO was higher than Cercon.

## 4. Discussion

The use of resin cement for luting restorations, especially all ceramic restorations, is quite common. Adequate polymerization of resin cement will result in high-bond strength between tooth and restoration. Light intensity is one of the most important factors that affects polymerization of light-cured resin cements. Recent studies have shown a positive correlation between light intensity and the degree of conversion of restorative materials [21–23]. Rueggeberg et al. [12] suggested that the adequate intensity for a light-curing unit is 400 mW/cm^2^ in order to initiate polymerization of resin-based material, but is unsuitable when the light-curing unit irradiated light with intensity under 233 mW/cm^2^. The ISO [24] also suggested a minimum intensity of 300 mW/cm^2^ in the 400–515 nm wavelength bandwidth. The LED light-curing unit used in this study provided light intensity up to 1,450 mW/cm^2^, as measured by a radiometer, and induced a high degree of polymerization and surface hardness of resin cements. However, when irradiating through ceramic discs, the light intensity decreased as a function of the type and thickness of the ceramics [25–27].

The dental ceramics used in this study were IPS e.max Press and Cercon, which are indicated for veneer and crown fabrication. The IPS e.max Press, a lithium disilicate glass ceramic, has 4 types corresponding to opacity: high translucency (HT), low translucency (LT), medium opacity (MO), and high opacity (HO). Cercon is one of the CAD/CAM zirconia ceramics possessing high strength and opacity [1]. In this study, when irradiating light through Cercon and IPS e.max Press, the light intensity through Cercon was lower compared with IPS e.max Press, which has lower opacity.

Surface hardness is one of the most effective methods to evaluate the polymerization of resin cement. Many studies have shown that Knoop hardness has a positive correlation with the degree of conversion of resin cement [15, 17]. Furthermore, Knoop hardness has also been reported to be related to the light intensity of the light-curing unit [20, 27]. Resin cement which received higher light intensity had better polymerization and higher Knoop hardness than that receiving lower light intensity. In this study, surface hardness was measured at the bottom surface of specimens, opposite to the light activation surface, to show the degree of polymerization of the whole material. A study by Aguiar et al. [28] has shown the surface hardness of the top surface was the highest. It decreased significantly moving from the top toward the bottom of the specimen due to greater distance from the light guide. Moreover, resin cement can disperse light from the light-curing unit as resin matrix and filler particle scatter the light and thus reduce light intensity when passing through resin cement [13, 29]. Consequently, the surface hardness of the top surface does not indicate the hardness of other portions or degree of conversion of materials.

Resin cement polymerized under ceramic discs received lower light intensity as the thicknesses and opacity of ceramic increased. The decrease of surface hardness was statistically significant as a function of change in those parameters [30]. This study found that direct activation resin cement had higher surface hardness than resin cement which was activated through ceramic, especially Variolink Veneer polymerized under Cercon 1.0, 1.5, and 2.0 mm. While both resin cements were polymerized under all ceramic types of 0.5 and 1.0 mm thickness, the surface hardness did not vary from the surface hardness of the control group except NX3 polymerized under IPS e.max Press HO 1.0 mm and Variolink Veneer polymerized under Cercon 1.0 mm. For ceramic thicknesses of 1.5 and 2.0 mm, the surface hardness was different from that of the control group where resin cement was polymerized under ceramic with higher opacity, such as IPS e.max Press HO and Cercon. This may imply that the thickness of ceramic has less effect on high-translucency ceramics than low-translucency or high-opacity ceramics. The surface hardness of resin cements polymerized under high-opacity ceramic was lower than those polymerized under lower-opacity ceramic. The surface hardness of both resin cements polymerized under Cercon zirconia ceramic showed the lowest hardness, while resin cement polymerized under IPS e.max Press HT, a glass ceramic, tended to have the highest hardness of all thicknesses. These results are in line with the finding of Borges et al. [31] who reported that resin cement polymerized under alumina and zirconia ceramic had lower hardness than cement polymerized under glass ceramic. Furthermore, the study found that NX3 had higher hardness than Variolink Veneer in all groups. These results are likely due to different compositions of resin cement such as types, quantities, and size of filler, which affect polymerization of materials [32, 33].

## Figures and Tables

**Table 1:** The mean (KHN) ± standard deviation of surface hardness of two resin cements when polymerized under different ceramic a.

Ceramic/thickness (mm)	KHN (mean ± SD)	
	Variolink Veneer	NX3
Control	21.64 ± 3.7^1^	30.28 ± 1.8^2^

e.max HT/0.5	19.39 ± 3.8^1Aa^	30.16 ± 1.8^2Ne^
e.max HT/1.0	18.39 ± 3.3^1ABb^	29.80 ± 2.2^2Nf^
e.max HT/1.5	17.16 ± 2.9^1ABc^	27.79 ± 1.9^2Nh^
e.max HT/2.0	15.38 ± 2.1^1Bd*^	24.83 ± 3.3^2Ok*^

e.max LT/0.5	19.42 ± 3.2^1Ca^	28.30 ± 2.3^2Pe^
e.max LT/1.0	18.40 ± 3.1^1CDb^	27.29 ± 2.3^2POfg^
e.max LT/1.5	16.45 ± 3.2^1CDc^	26.90 ± 2.1^2QRhi*^
e.max LT/2.0	15.30 ± 3.2^1Dd*^	22.99 ± 2.1^2Rk*^

e.max MO/0.5	19.43 ± 3.2^1Ea^	28.46 ± 1.5^2Se^
e.max MO/1.0	17.97 ± 3.2^1EFb^	27.45 ± 2.2^2Sfg^
e.max MO/1.5	16.47 ± 3.3^1EFc^	26.31 ± 1.8^2Shi*^
e.max MO/2.0	14.81 ± 2.8^1Fd*^	23.81 ± 2.4^2Tkm*^

e.max HO/0.5	19.81 ± 3.9^1Ga^	29.48 ± 2.6^2Ue^
e.max HO/1.0	18.15 ± 3.4^1GHb^	27.16 ± 2.4^2UVfg*^
e.max HO/1.5	16.34 ± 2.8^1HJc*^	25.51 ± 2.3^2Vhi*^
e.max HO/2.0	13.01 ± 2.1^1Jd*^	20.08 ± 2.6^2Wkm*^

Cercon/0.5	17.66 ± 3.3^1Ka^	28.80 ± 2.3^2Xe^
Cercon/1.0	16.73 ± 2.8^1KLb*^	27.11 ± 2.5^2XYg*^
Cercon/1.5	14.47 ± 2.4^1LMc*^	24.70 ± 2.7^2Yj*^
Cercon/2.0	13.15 ± 3.1^1Md*^	21.82 ± 2.4^2Zm*^

Different numbers in the same row indicate significant difference (*P* < 0.05) between 2 resin cements. Different uppercase letters in the same column indicate significant difference (*P* < 0.05) among ceramic thicknesses in each ceramic type.

Different lowercase letters in the same column indicate significant difference (*P* < 0.05) among ceramic types in each ceramic thickness.

*Indicates significant difference (*P* < 0.05) from control group.
